# Impact of implemented vaccination strategies on vaccine uptake and attitude of final year pharmacy students toward COVID-19 vaccines in Gezira State, Sudan

**DOI:** 10.1016/j.jvacx.2023.100416

**Published:** 2023-11-28

**Authors:** Alhumaira Wedaa, Mohamed Elmustafa, Hanaa A Babiker, Rana Ahmed

**Affiliations:** a Department of Pharmacology, Faculty of Pharmacy, University of Gezira, Wad Medani, Sudan; b Wad Medani College of Medical Sciences and Technology, Wad Medani, Sudan; c The Epidemiological Laboratory, Khartoum, Sudan

**Keywords:** COVID-19, Vaccine, Vaccination uptake, Hesitancy, Supportive-strategies

## Abstract

Vaccine hesitancy is a global threat to public health. Hesitant individuals pose a major concern, as they can be viewed as a source of infection, which may lead to another outbreak. Effective strategies are needed to increase uptake, decrease hesitancy, and achieve herd immunity. This study aims to identify the impact of implemented strategies on COVID-19 vaccine hesitancy and uptake among final-year pharmacy students, and their acceptance and attitudes towards mandatory COVID-19 vaccination. An anonymous, internet-based cross-sectional study was developed using Google Forms and administered to final-year pharmacy students (254) at all pharmacy colleges in Wad Medani city, Sudan between August and September 2022. Overall, 30.7 % of students were hesitant to get the COVID-19 vaccine. The majority of students, 69.3 %, were already vaccinated and 60.9 % of them were initially hesitant about getting the vaccine but eventually did so. Receiving the COVID-19 vaccine was significantly associated with the institution students attended (p < 0.001). Institutions that had implemented encouraging vaccination strategies had a higher percentage of vaccinated students: 84.2 % and 77.1 %, compared to the institution that did not adopt any vaccination strategies 28.3 %. Availability of COVID-19 vaccines to students (OR 1.67 CI (0.70–3.96)), and encouraging COVID-19 vaccination in a way close to mandatory (OR 4.29, CI (1.85–9.96)) had the highest odds in increasing the vaccination uptake. While, not implementing any vaccination strategy (OR 0.24, CI (0.07–0.85) was less likely to increase vaccination uptake. Also, it was found that 72.5 % of students would accept mandatory vaccination for COVID-19. This study provides policymakers with evidence-based strategies that could increase the uptake and decrease hesitancy toward COVID-19 vaccines among a group of university students. Policymakers should encourage all universities to provide COVID-19 vaccines to their students, either through clinics or vaccination campaigns, and consider mandating the COVID-19 vaccine.

## Introduction

The main preventive measure to overcome the COVID-19 pandemic is developing and administering effective, safe, and affordable vaccines to decrease hospitalization, and the severity of the disease, and to establish herd immunity [1]. To achieve herd immunity, vaccination of 75–90 % of the population with a vaccine that has (80 %) efficacy is needed, and the whole population if the vaccine has a lower efficacy [2]. A world health organization goal set in 2021 was to fully vaccinate 70 % of the population in each country by mid-2022, but Sudan didn't meet that target [3]. Similarly, this target is considered unrealistic for some African countries because there are many barriers that need to be addressed and solved [4]. In Sudan, 3.8 % of the Sudanese people were partially vaccinated, and 28.6 % were fully vaccinated as of 2 April/2023 [5].

Vaccine hesitancy is a rate-limiting factor in the success of vaccination campaigns [6]. It is considered by the WHO one of the top ten threats to global health [7], and is defined as a delay in acceptance or refusal of vaccination despite the availability of vaccination services [8]. The low vaccination rate and delay in vaccination acceptance has the potential to prolong the pandemic and allow new variants to develop continuously [9]. Despite the nature of the medical profession, vaccine hesitancy has found its way among healthcare workers [10], [11] and medical students [12], [13], [14], [15], [16], [17]. The level of hesitancy toward COVID-19 vaccine was found to be 18.9 % among medical students in 39 countries [12], 14 % in Western New England University [15], 45.7 % among Egyptian medical students [16], and 44.2 % among Sudanese medical students [13]. This puts more pressure on the scientific community and policy makers to develop strategies to increase uptake and decrease hesitancy among such important groups of the population. This can be achieved by providing accessible evidence-based information addressing deterrents of vaccination since many countries did not implement mandatory vaccinations [18]. Educational campaigns are apparently one way forward, but may not be sufficient alone; they should be combined with other encouraging strategies [19]. Using multiple strategies has been shown to be the most effective way to boost vaccination uptake. This is because of the complexity of vaccine hesitancy which cannot be overcome by a single strategy [19], [20], [21], [22], [23].

To increase vaccination rates among students, five strategies have been proposed: educating students with accurate information, increasing vaccine access through vaccination clinics, updating the curriculum, encouraging student participation in vaccination campaigns, and requiring mandatory vaccinations [22]. Several universities are mandating vaccination to ensure safety, reduce transmission, return to normal educational activities, and decrease testing [24]. Mandatory vaccination in different universities was found to be very effective in both reducing the cases of COVID-19 and increasing the uptake [25].

In many countries, the most accessible health worker is the pharmacist [26], so the public turns to them for advice and reliable information [27]. Pharmacy partnerships during the COVID-19 pandemic helped increase the uptake of vaccines by making them more accessible to faraway areas and vulnerable populations [28]. When pharmacists act as vaccinator, vaccine access increases and the time to reach 80 % first dose coverage decreases [29]. During the pandemic, pharmacists have adopted a major role in minimizing vaccine hesitancy and increasing the uptake of vaccines through educating patients, increasing awareness and illustrating the benefits of vaccination [30], [31]. This picture has not been reflected in pharmacists, nor in healthcare students in Sudan, and several studies have demonstrated high levels of hesitancy among them [10], [13]. The final-year pharmacy student must be prepared for all future responsibilities ranging from simple education to precise immunization activities. It is, therefore, important to investigate the vaccination acceptance of graduates who will become future practitioners.

Since the COVID-19 pandemic affects all communities differently, it is critical to identify effective strategies for promoting the uptake of the COVID-19 vaccine on each community. Developing and disseminating best practices and lessons learned should be encouraged regularly [32]. All possible strategies to decrease vaccine hesitancy must be considered as it is a very complex and dynamic phenomenon [32], [33]. Many studies have investigated vaccine acceptance and hesitancy among university students [12], [17], [34], [35], but only a few have highlighted strategies that could increase the uptake of COVID-19 vaccines and decrease hesitancy. Therefore, the role of vaccination-supportive strategies on increasing uptake of COVID-19 vaccines needs to be explored [36]. Our study aims to investigate the impact of various strategies in promoting COVID-19 vaccination among final-year pharmacy students. The goal is to provide policymakers with real-life implemented strategies that could increase vaccine uptake and decrease hesitancy toward the COVID-19 vaccine. Our hypothesis is that institutions that have implemented vaccination-supportive strategies will have a significantly higher percentage of vaccinated final-year pharmacy students compared to institutions that have not adopted any such strategies.

## Methods

### Study design and setting

This was an online-based cross sectional study targeting the entire population of final year pharmacy students in Wad Medani City, the capital of Gezira State in Sudan. Four institutions award a bachelor of pharmacy degree in Wad Medani, one public university, and three private colleges. One private institution was excluded from the study as they did not have pharmacy students in their final year at the time of the study. Two institutions adopted encouraging vaccination strategies: provided vaccines through clinics or vaccination campaigns, encouraged vaccination in a way close to mandatory, implemented educational and awareness events about COVID-19 and vaccination, and sent periodic reminders to encourage students to get their vaccines. On the contrary, one institution did not implement any official strategies to encourage students to receive COVID-19 vaccination.

### Study participants

All final-year pharmacy students at the three study sites were invited to participate in the survey. Each student was given the option to refuse participation or to withdraw from the study at any time during data collection if they did not wish to complete the questionnaire.

### Sample size

The total number of student population was 254. We anticipated to sample 154 participants (60 % of the target population). Sample size was calculated using Raosoft [37] with a 5 % margin of error, 95 % confidence level and 50 % response distribution. Provision of internet service at the time of data collection, sensitization of students and provision of backup paper-based questionnaires were implemented to increase the response rate.

### Study tool and data collection

The questionnaire (12 questions) was developed using online Google Forms after an extensive literature review [10], [12], [13], [15], [16] and expert input. Demographic data, vaccination status, decision about vaccination, factors that encouraged vaccination among students, awareness of students of the adopted strategies, and acceptance and attitudes toward mandatory vaccination were collected from participating students (Supplement 1). We selected factors affecting vaccination uptake, encompassing concerns about safety, the desire to protect others, to reduce restrictions, availability of vaccines, to decrease complications, and encouragement from others [10], [12], [13], [16], [38], [39]. A pilot study was conducted among 29 final-year pharmacy students studying outside the study area. Based on the results, we determined the face and content validity of the questionnaire, internal consistency was tested (Cronbach’s alpha = 0.7), and irrelevant, ambiguous, and unclear questions were removed. The questionnaire was translated to Arabic and back-translated to English by a professional translator. A presentation about the study was conducted with students in each faculty/college to sensitize them, encourage participation, and explain the survey questions prior to data collection. On the same day, the survey link was distributed through the existing WhatsApp® group that students use for communication. The data was collected between August and September 2022.

### Statistical analysis

Data were entered into Microsoft Excel (Microsoft Corporation Washington, USA) for cleaning and coding. The analysis was conducted using IBM Statistical Package for the Social Sciences (SPSS, IBM Corporation, Armonk, New York, USA) version 25. Descriptive statistics were performed on all participant characteristics. Categorical variables were presented as absolutes, percentages, and frequencies. Questions with multiple answers were analyzed as multiple response questions. A chi-square test was used to identify associations between demographic data and vaccination uptake. It was also used to identify factors that were associated with receiving the COVID-19 vaccine, and decisions and attitudes about vaccination. All statistical tests were performed at 95 % confidence level and p-values < 0.05 were considered significant. To assess the effect size, Cohen's d statistic was reported. Univariable and multivariable logistic regression analyses were used to identify association between receiving the COVID-19 vaccine and adopted strategies and acceptance and attitude toward mandatory vaccination. Age and gender were forced in all models. All other variables with a p-value < 0.05 from univariable analysis were included in multivariable analysis. Adjusted odds ratios (OR) and confidence intervals (CI) were presented.

### Ethical considerations

Ethical approval was obtained from the health sector research ethics committee at the University of Gezira (No: 24-22). All participants provided informed written consent before they could access the online survey and had the option to withdraw at any time during the survey. Respondents’ anonymity and confidentiality of information was assured and maintained and no identifying data were collected from students.

## Results

### Participant characteristics

A total of 254 registered final- year pharmacy students at the included institutions were approached at the time of the study. These were 118, 82 and 54 students at institutions 1, 2, and 3 respectively.

In total, 218 students completed the Survey, giving a response rate of 85.8 %. The individual response rate was 81.35 %, 92.6 %, and 85.1 % for institutions 1, 2 and 3 respectively. The majority of participants 76.1 % (n = 166) were between the ages of 21 and 24, and 73.4 % (n = 160) were females. Most participants (n = 157, 72 %) were unmarried, 67.4 % (n = 147) were from a city/town, and 86.7 % (n = 189) did not have a parent working in a health-setting (Table 1).

**Table 1:** Characteristics of participants who received or did not receive the COVID-19 vaccine (n = 218).

Variables		Received a COVID-19 vaccine N (%)		P-value	Cohen's d (CI)
		No	Yes		
Age groups	21–24	42 (62.7 %)	124 (82.1 %)	0.004	0.39 (0.10–0.68)
	25–28	22 (32.8 %)	24 (15.9 %)		
	29–32	3 (4.5 %)	1 (0.7 %)		
	33–36	0 (0.0 %)	2 (1.3 %)		
Gender	Male	25 (37.3 %)	33 (21.9 %)	0.017	−0.35(-0.64- −0.063)
	Female	42 (62.7 %)	118 (78.1 %)		
Marital status	Single	40 (59.7 %)	117 (77.5 %)	0.059	0.35 (0.06–––0.64)
	Married	13 (19.4 %)	16 (10.6 %)		
	Engaged	13 (19.4 %)	16 (10.6 %)		
	Divorced	1 (1.5 %)	2 (1.3 %)		
Originally from?	Village	24 (35.8 %)	47 (31.1 %)	0.495	−0.10(-0.39–––0.19)
	City/town	43 (64.2 %)	104 (68.9 %)		
Do any of your parents work in a health setting?	Yes	11 (16.4 %)	18 (11.9 %)	0.367	−0.13(-0.42–0.16)
	No	56 (83.6 %)	133 (88.1 %)		

P- value: Chi-square test, CI = Confidence Interval.

### Vaccination hesitancy and uptake

**Fig. 1:**
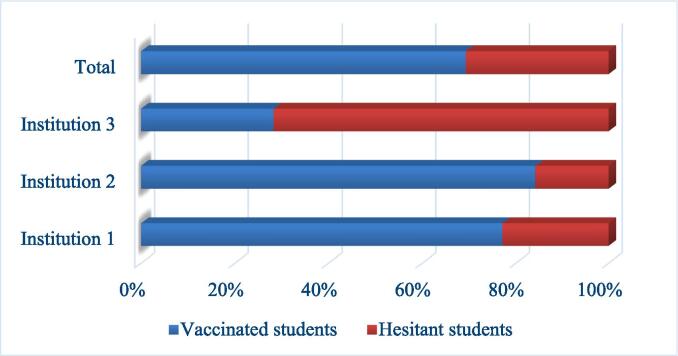
COVID-19 vaccine hesitancy and uptake among pharmacy students in each institution (n = 218).

In this study the majority of students 69.3 % (n = 151) were vaccinated, while 30.7 % (n = 67) were hesitant. At institution 1, 77.1 % (n = 74) of students took at least one dose of the vaccine, and 22.9 % (n = 22) were hesitant. In institution 2, 84.2 % (n = 64) of students received at least one dose of the vaccine and 15.8 % (n = 12) were hesitant. Only 28.3 % (n = 13) of the students at institution 3, had received at least one dose of the vaccine and 71.7 % (n = 33) were hesitant (Fig. 1). Chi-Square analysis revealed a significant difference in receiving the COVID-19 vaccine among students from the different institutions (p < 0.001).

### Vaccinated students

Regarding attitude toward vaccination decisions, 60.9 % of vaccinated students (n = 92) were not willing to receive the vaccine at the beginning of the pandemic, but changed their minds over time, while 39.1 % (n = 59) were willing to receive the vaccine from the start.

The main five factors that encouraged students to be vaccinated as reported by participants were the protection of colleagues, family, and friends 57.6 % (n = 87), to decrease the complications and severity of the disease if infected with COVID-19 54.3 % (n = 82), the availability of the vaccine at their university 37.1 % (n = 56), to be less worried about COVID-19 26.5 % (n = 40), and as a traveling requirement 24.5 % (n = 37).

**Fig. 2:**
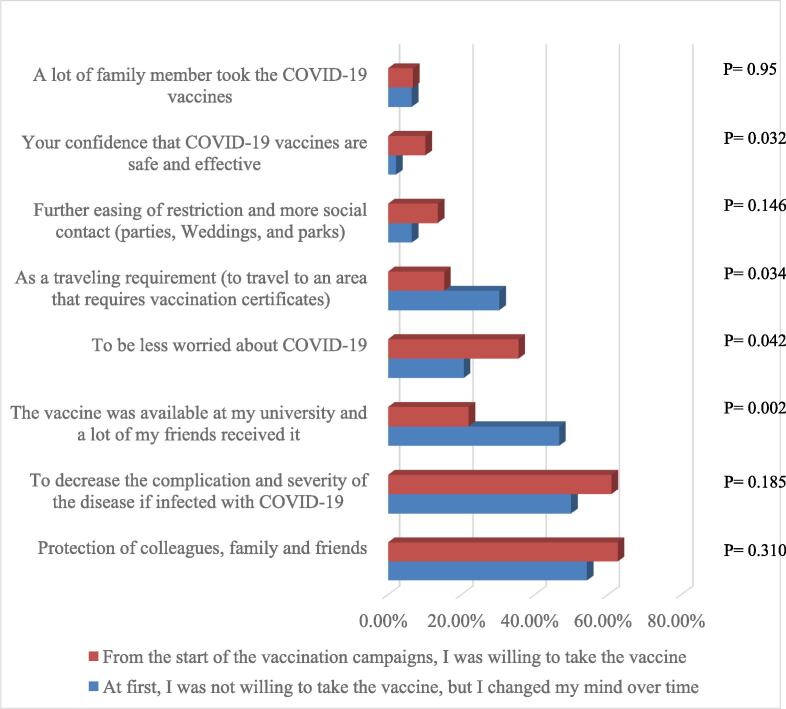
Association between vaccination decision attitudes and factors that encouraged vaccinated students to receive the COVID-19 vaccine using Chi-square test (n = 151).

Among vaccinated students, the distribution of the encouraging factors among students who initially refused the vaccine, but later changed their mind and students who were willing to receive the vaccine from the beginning was almost the same except in four factors that were found to be statistically associated with vaccination decision attitudes through using the Chi-square test. These factors were: traveling requirements (p = 0.034), to be less concerned about COVID-19 infection (p = 0.042), confidence that the vaccine is safe and effective (p = 0.032), and availability of the vaccine at their university (p = 0.002), as shown in Fig. 2.

### The impact of different implemented strategies on receiving the COVID-19 vaccine

**Fig. 3:**
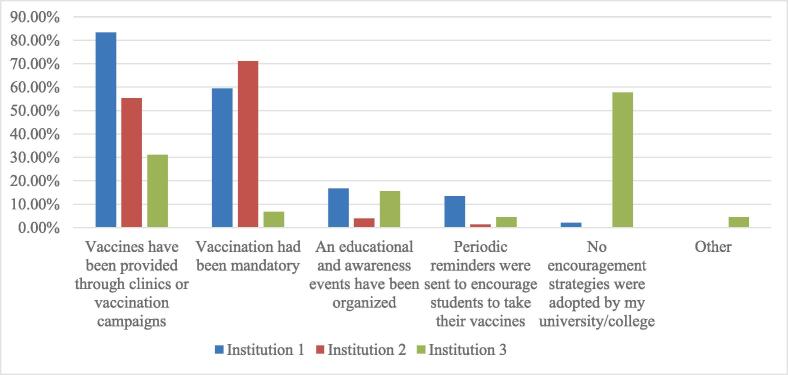
Awareness of students of the types of adopted strategies at their institutions (n = 218).

Institution 1 and 2, which adopted vaccination supportive strategies, had higher vaccination uptake and lower hesitancy levels compared to institution 3, which didn’t adopt any vaccination strategy (Fig. 1). To find out if the students were aware of implemented strategies, they were asked about what strategies had been implemented by their respective institutions. Students from institutions 1 and 2 knew that vaccines had been provided through clinics or vaccination campaigns in 83.3 % (n = 80) and 55.3 % (n = 42), respectively. Additionally, 71.1 % (n = 54) of students from institution 2, and 59.4 % (n = 57) of students from institution 1 understood that the vaccine was encouraged in a way close to mandatory. Furthermore, 57.8 % (n = 26) of students from institution 3 indicated that their college did not adopt any vaccination supportive strategies (Fig. 3).

In multivariable analysis, implementing an encouraging vaccination strategy close to mandatory had the higher odds in increasing vaccine uptake (OR 4.29, CI (1.85–9.96)). On the other hand, not employing any strategies and sending periodic reminders were less likely to increase vaccination uptake (OR 0.24, CI (0.07–0.85) & OR 0.18, CI (0.05–0.72)) respectively. Moreover, providing vaccines through clinics or campaigns had the higher odds in increasing vaccine uptake (OR 1.67 CI (0.70–3.96)). These results are further detailed in Table 2.

**Table 2:** Multivariable association between receiving the COVID-19 vaccine and the awareness of students of the adopted strategies by their institutions (n = 218).

Awareness of adopted vaccination strategies	Have you received a COVID-19 vaccine?(n = 218)		OR(CI)
	No n = 67	Yes n = 151	
Vaccination is mandatory	17 (25.4 %)	97 (64.2 %)	4.29*(1.85–9.96)
No encouragement strategies were adopted by my institution	21 (31.3 %)	7 (4.6 %)	0.24*(0.07–0.85)
Vaccines are provided through clinics or vaccination campaigns	35 (52.2 %)	101 (66.9 %)	1.67*(0.70–3.96)
Periodic reminders are sent to encourage students to take their vaccines	7 (10.4 %)	9 (6.0 %)	0.18*(0.05–0.72)
Educational awareness events are organized at our institution	7 (10.4 %)	19 (12.6 %)	1.54(0.48–5.01)

*p < 0.05., OR = Odds Ratio, CI = Confidence Interval.

### Acceptance of a mandatory vaccination strategy

The overall acceptance rate of mandatory vaccination was found to be 72.5 % (n = 158), as follows: 50.9 % will accept mandatory vaccination and will encourage other students to accept it and 21.6 % will accept it but will not encourage others. On the other hand, 11.5 % (n = 25) of students refused the idea of mandatory vaccination and would protest against it if enforced. In addition, 25.7 % (n = 56) considered it a violation of their privacy and human rights, and 1.4 % (n = 3) said they will consider the option of leaving the faculty if mandatory vaccination was put in place. Among unvaccinated students, 28.4 % (n = 19), said they will accept mandatory vaccination and will encourage others to accept it, and 14.9 % (n = 10), said they will accept it but will not encourage others to accept it. In contrast, 43.3 % (n = 29), of them considered it as a violation to their privacy. The odds of acceptance of mandatory vaccination that is associated with encouraging other students was significantly higher among vaccinated students when compared with unvaccinated ones (OR 14.28, CI (3.31–61.54). Also, those who will accept the mandatory vaccination but will not encourage other students had the higher odds of vaccination uptake (OR 8.65, CI 2.03–36.84). No other associations were observed between vaccination uptake and acceptance attitudes according to multivariable regression. See Table 3.

**Table 3:** Multivariable association between receiving the COVID-19 vaccine, and acceptance and attitudes of final year pharmacy students toward mandatory vaccination (n = 218).

Acceptance and attitudes toward mandatory vaccination	Have you received a COVID-19 vaccine?		Total	OR (CI)
	No	Yes		
You will accept it and encourage other students to accept it	19 (28.4 %)	92 (60.9 %)	111 (50.9 %)	14.28*(3.31–61.54)
You will accept it but you will not encourage other students to accept it	10 (14.9 %)	37 (24.5 %)	47 (21.6 %)	8.65*(2.03–36.84)
You will not accept it and will protest against it	14 (20.9 %)	11 (7.3 %)	25 (11.5 %)	1.98(0.55–7.17)
You will consider it as violation to your privacy and human rights	29 (43.3 %)	27 (17.9 %)	56 (25.7 %)	1.41(0.41–4.86)
You will consider the option of leaving the faculty	2 (3.0 %)	1 (0.7 %)	3 (1.4 %)	0.62(0.04–11.10)

*p < 0.05, OR = Odds Ratio, CI = Confidence Interval.

## Discussion

In order to understand the impact of vaccination supportive strategies on COVID-19 vaccine uptake among university students, this study attempted to investigate hesitancy and acceptance of COVID-19 vaccines in two academic institutions that implemented vaccination supportive strategies in comparison with one that did not. We focused on the final year pharmacy students because of their crucial, near future, role in improving public health and encouraging vaccination against COVID-19. These roles range from simple education activities to precise immunization activities that are designed to increase the uptake of vaccines and reduce hesitancy among healthcare workers and the general population [29], [31].

The study found that 69.3 % of participating students were vaccinated, while 30.7 % were hesitant. The hesitancy level found in this study is higher than that found among medical students in 39 countries 18.9 % [12], and among pharmacy students in Western New England University 14 % [15]. On the other hand, it is lower than the 44.2 % hesitancy shown among medical students in Sudan [13], and the 45.7 % shown among Egyptian students [16]. There is a wide variation in hesitancy and vaccination rates among university students and this can be attributed to different reasons including that hesitancy changes over time and place [8] and adoption of strategies to increase the uptake similar to those shown in our results.

It is worth noting that the majority (60.9 %) of vaccinated students were not willing to receive the vaccine at the beginning of the pandemic, but changed their minds over time. Change of attitudes towards COVID-19 vaccines has been reported in a survey conducted across England and Wales as 86 % of respondents changed their minds toward accepting the COVID-19 vaccines [40]. Similar findings were also observed in China where willingness to receive the vaccines had increased from 66.6 % to 90.5 % [41]. Change of attitudes doesn't have to be in the direction of accepting the vaccine; sometimes it's the opposite. A study conducted in China found the acceptance of vaccine dropped from 44.2 % to 34.8 % [38]. Al-Mulla and his colleagues highlighted this point when they noticed that despite all the vaccination efforts there was still a persistent hesitancy [42]. Therefore, it is crucial to identify factors that influenced students' decision-making and develop early effective initiatives to increase the uptake, awareness, and trust in COVID-19 vaccines as fast as possible because of the dynamic nature of vaccine hesitancy. This study found a statistically significant association between vaccination decision attitudes and the vaccine's availability on campus. This association raises an insightful point: the majority of students changed their vaccination decision toward accepting the vaccine potentially as a result of vaccination-supportive strategies at their institutions particularly as a result of availability and ease of access to the vaccines.

The different percentages of vaccinated students at the three study sites (77.1 %, 84.2 % and 28.3 %) show the significant implications of adopted strategies in increasing the uptake and decreasing hesitancy levels. Two of the study institutions [1,2] adopted strict and encouraging strategies to increase the uptake of the COVID-19 vaccines. They also made the COVID-19 vaccines available through the clinic or vaccination campaigns on campus. In contrast, the third institution did not announce any strategy to encourage vaccination among students. Our results showed a high odds ratio between vaccination uptake and the awareness of providing vaccines through clinics/campaigns. This is in alignment with a study conducted by Jarrett et al. who reported making the vaccine available as one of the most effective methods for increasing vaccine uptake and reducing vaccine hesitancy [20]. This strategy was also used by a Brazilian hospital in 2006 to boost the uptake of the influenza vaccine by making the vaccine freely accessible to healthcare professionals in the area. The vaccination rate increased from 6 % to 45 % [43]. Vaccine accessibility is considered to be one of the major barriers to vaccination [31], [44], [45]. According to a study conducted in Gezira State, Sudan, inaccessibility to the COVID-19 vaccine was the primary reason for the delay in vaccination uptake [36]. To overcome this barrier, the general population should be informed about the availability of vaccines [36], using mobile units [44], and guided to community pharmacies as vaccination centers [31]. Hence, it is prudent that other institutions follow suit in making COVID-19 vaccines available in close proximity to beneficiaries.

The second strategy used by institutions 1, and 2 was adopting an encouraging strategy that is close to mandatory which has proven effective in increasing COVID-19 vaccination uptake. This is in alignment with a study conducted in 2022 which compared 94 higher educational institutions from 10 different states in the United States to assess the impact of mandatory vaccination. Institutions with mandatory vaccinations showed a higher vaccination rate, suggesting that a mandatory vaccination strategy is one of the most effective strategies to increase vaccination rates [25]. Moreover, mandating vaccination is considered one of the most effective methods that could increase the uptake by more than 25 % [20]. Students in our study viewed the strict vaccination strategy adopted by two of the studied institutions as mandatory vaccinations. However, this was not the case as they were in fact motivation announcements. Unvaccinated students were not screened or prevented from accessing the university or college admission as a result of not being vaccinated. This strategy was also found to be effective and was significantly associated with vaccination uptake. An online survey conducted among Australian university students revealed that 79.4 % supported mandatory vaccination, but only 38.9 % agreed that unvaccinated students or staff should lose their position. In other words, high acceptance of the mandatory vaccination strategy does not necessarily equate to acceptance of its consequences [32]. We assume, if organized, well-designed strict encouraging strategies or mandatory vaccination are implemented in collaboration with different authorities, there is a greater likelihood of an increase in vaccination uptake and decrease in hesitancy levels.

Regarding the acceptance and attitudes toward adopting mandatory vaccination, our findings indicate that most students accept the COVID-19 vaccine to be mandated at their university/college. There is some similarity between this finding and that reported in previous studies among university students [17], [34], [35]. Likewise, similar results were found regarding acceptance of mandatory influenza vaccination [46], [47]. In light of our findings, policymakers can consider requiring COVID-19 vaccinations for all university students in Sudan as a preliminary step toward implementing mandatory vaccination strategies. In addition to the need for a large-scale study, this strategy should be implemented with caution. This is because 25.7 % of students view it as a violation of their privacy and human rights, 11.5 % will protest against it, and 1.4 % will consider leaving the university/college in the case of COVID-19 vaccination being mandatory. In addition to the announcement of mandatory vaccination; implementation, design, availability of vaccines, encouragement, and convincing of the target population play a critical role in the success of such strategies [48]. As hesitant individuals pose a major threat to the health of society, they can be viewed as sources of infection, leading to another outbreak. Dhama and his colleagues proposed requiring vaccination of all populations, regardless of ethnicity or nationality [49]. Also, using different strategies were recognized to be more effective than one method approach in decreasing hesitancy [19], [20], [21].

### Strengths and limitations

To our knowledge, this is the first research of its kind in Sudan that attempted to highlight the vaccination strategies, and the acceptance of mandatory vaccination among university students. The study provides significant information about the impact of different vaccination strategies that are employed by the students' respective institutions on vaccine hesitancy and uptake. In addition, our findings give valuable insights into acceptance and attitudes toward mandatory vaccination against COVID-19. Furthermore, with a response rate of 85.8 %, our study can be deemed to have achieved a relatively high level of participation.

This study has some limitations. It only targeted final year pharmacy students in Wad Medani, thus generalization and extrapolation should be interpreted with caution for other parts of Sudan and other sub group populations, as well as beyond Sudan. Due to the anonymous nature of the online questionnaire, we are unable to tell whether students have completed the questionnaire more than once.

## Conclusions

Implementing vaccination-supportive strategies increased the level of vaccination uptake and decreased the level of hesitancy among students. The majority of vaccinated students were hesitant at first, but changed their minds over time potentially as a result of vaccination-supportive strategies at their institutions particularly as a result of availability and ease of access to the vaccines. Most students would accept a mandatory vaccination strategy. Nevertheless, a quarter of students believe this strategy violates their privacy and human rights. Collectively, these findings provide an insight into strategies that could increase the uptake and decrease hesitancy and thus allow policymakers to implement effective vaccination campaigns built on evidence-based strategies. Therefore, we recommend that stakeholders devise plans to ensure vaccine availability in every educational institution and consistently encourage students to get vaccinated.

## Author contributions

**Alhumaira Wedaa**: Conceptualization, designing of the project, data collection, data analysis, and Writing – original draft.

**Mohamed Elmustafa**: Designing the project, supervision of data collection and analysis, writing and critically reviewing the manuscript.

**Rana Ahmed:** Participation in data analysis and interpretation of the data, writing and critically reviewing the manuscript.

**Hanaa A**. **Babiker:** Participation in the interpretation of the data, writing and critically reviewing the manuscript.

All authors approved the final manuscript of the article and attest they meet the ICMJE criteria for authorship.

## Funding

This research did not receive any specific grant from funding agencies in the public, commercial, or not-for-profit sectors.

## CRediT authorship contribution statement

**Alhumaira Wedaa:** . **Mohamed Elmustafa:** . **Hanaa A Babiker:** Investigation, Resources, Validation, Writing – review & editing. **Rana Ahmed:** .

## Declaration of competing interest

The authors declare that they have no known competing financial interests or personal relationships that could have appeared to influence the work reported in this paper.

## Data Availability

Data will be made available on request.
