# Supplementary material for: Impact of implemented vaccination strategies on vaccine uptake and attitude of final year pharmacy students toward COVID-19 vaccines in Gezira State, Sudan
Source: Vaccine X. 2023 Nov 28;15:100416. doi: 10.1016/j.jvacx.2023.100416 (PMC10701370; doi:10.1016/j.jvacx.2023.100416)
Supplement: Supplementary Data 1 [file mmc1.pdf]

Impact of implemented vaccination strategies on vaccine uptake and attitude of final year pharmacy students toward COVID-19 vaccines in Gezira State, Sudan.

This is a survey using Google form targeting final-year pharmacy students in different faculties of pharmacy in Wad-Medani, Gezira State, Sudan. Our general objective is to explore the impact of implemented vaccination strategies on vaccine uptake
* All your answers will be kept confidential and anonymous.
* There is no risk associated with completing this survey.
* You will need about 5 minutes to complete this questionnaire.

* Indicates required question

1. you are invited to participate in this research. By clicking (I agree) you are indicating you are a final year pharmacy Student studying in Wad Medani, Gezira State and you agree to participate in this research by completing this online questionnaire *

Mark only one oval.

- I agree
- I disagree

Part 2

demographic and basic information

2. Currently, you are a final-year pharmacy student at which university or college *

Mark only one oval.

- Institution 1
- Institution 2
- Institution 3

3. Age *

____

4. Gender *

Mark only one oval.

- Male
- Female

5. Marital status *

Mark only one oval.

- Single
- Married
- Engaged
- Divorced
- Widow/Widower
- Other: _______________

6. Where are you originally from? *

Mark only one oval.

- Viliage
- City/town

7. Do any of your parents work in a health setting? *

Mark only one oval.

- Yes.
- No.

Part 3

vaccine uptake and hesitancy

8. Have you received a COVID-19 vaccine? *

Mark only one oval.

- ◯ Yes
- ◯ No *Skip to question 11*

part 4

Vaccinated Students

9. In regards to your vaccination decision, which status best describes your attitude? *

Mark only one oval.

- ◯ From the start of the vaccination campaigns, I was willing to take the vaccine.
- ◯ At first, I was not willing to take the vaccine, but I changed my mind over time.

10. Which factors or reasons encouraged you to get the vaccine? *
You can select more than one option

Check all that apply.

- [ ] To decrease the complication and severity of the disease if infected with COVID-19.
- [ ] Protection of colleagues, family and friends.
- [ ] Further easing of restriction and more social contact (parties, Weddings, parks,..etc).
- [ ] As a traveling requirement (to travel to an area that requires vaccination certificates).
- [ ] To be less worried about COVID-19.
- [ ] Your confidence that COVID-19 vaccines are safe and effective.
- [ ] A lot of family member took the COVID-19 vaccines.
- [ ] The vaccine was available at my university and a lot of my friends received it.
- [ ] Other: ____________________

Skip to question 11

part 5

Strategies

11. In order to encourage vaccination, what strategies have been implemented by your university /college? *
You can select more than one option

Check all that apply.

- [ ] Vaccines have been provided through clinics or vaccination campaigns.
- [ ] Vaccination had been mandatory .
- [ ] An educational and awareness events have been organized.
- [ ] Periodic reminders were sent to encourage students to take their vaccines.
- [ ] No encouragement strategies were adopted by my university/college.
- [ ] Other: ____________________

12. Suppose your faculty decided to adopt a new policy that mandate all students to receive COVID-19 vaccines (proof of vaccination would be required for entry): *
You can select more than one option

Check all that apply.

- [ ] you will accept it and encourage other students to accept it.
- [ ] you will accept it but you will not encourage other students to accept it.
- [ ] You will not accept it and will protest against it.
- [ ] You will consider it as violation to your privacy and human rights.
- [ ] You will consider the option of leaving the faculty.
- [ ] Other: ____________

13. Thank you very much for your participation. *

____

This content is neither created nor endorsed by Google.

Google Forms
